# Nomogram for predicting central lymph node metastasis in T1-T2 papillary thyroid cancer with no lateral lymph node metastasis

**DOI:** 10.3389/fendo.2023.1112506

**Published:** 2023-01-19

**Authors:** Yubo Sun, Wei Sun, Jingzhe Xiang, Hao Zhang

**Affiliations:** Department of Thyroid Surgery, The First Hospital of China Medical University, Shenyang, China

**Keywords:** SEER (Surveillance Epidemiology and End Results) database, T1-T2, papillary thyroid carcinoma (PCT), central lymph mode metastasis, lateral lymph node metastasis, nomogram

## Abstract

**Objective:**

Whether routine central lymph node dissection (CLND) is necessary for T1-T2 papillary thyroid carcinoma (PTC) patients without certain lateral lymph node metastases (LLNM) remains controversial. This study aims to construct a nomogram that predicts central lymph node metastasis (CLNM) for T1-T2 PTC patients without LLNM.

**Methods:**

We retrospectively reviewed adult T1-T2 PTC patients with no LLNM retrieved from the Surveillance, Epidemiology, and End Results (SEER) database from 2010 to 2015. We also collected data from patients treated at the First Hospital of China Medical University between February and April 2021 for external validation. Logistic regression model was used to construct a risk prediction model nomogram. The receiver-operating characteristic (ROC) curve, calibration plot, and decision curve analyses (DCA) were used for assessing the nomogram.

**Results:**

5,094 patients from the SEER database and 300 patients from our department were finally included in this study. Variables such as age, gender, race, tumor size, multifocality, and minimal extrathyroidal extension (mETE) were found to be associated with CLNM and were subsequently incorporated into our nomogram. The C-index of our constructed model was 0.704, while the internal and external validation C-indexes were 0.693 and 0.745, respectively. The nomogram was then evaluated using calibration and decision curve analyses.

**Conclusion:**

A visualized nomogram was successfully developed to predict CLNM in T1-T2 PTC patients without LLNM and assist clinicians in making personalized clinical decisions.

## Introduction

The incidence of papillary thyroid cancer (PTC), the most common type of differentiated thyroid cancer (DTC), has increased over the last decades (1, 2). The use of high-resolution ultrasonography (US) and fine needle aspiration (FNA) biopsy techniques have led to an increase in the diagnosis of PTC (3). However, the sensitivity of US in assessing deep anatomical spaces of the central neck compartment is quite low (4).

In 2015 the American Thyroid Association Guidelines recommended against prophylactic central lymph node dissection (PCLND) for small (T1-T2), non-invasive, clinically node-negative (cN0) PTC, while PCLND could be considered for patients with T3-T4 or cN1b (5). However, the Chinese Thyroid Association recommends routine central lymph node dissection (CLND) for all PTC patients to stage disease and prevent recurrence (6). Thus, the routine CLND for T1-T2 patients without certain lateral lymph node metastases (LLNM), comparing to T3-T4 or cN1b patients, remains controversial. In the 8^th^ edition of American Joint Committee on Cancer (AJCC) TNM classification, PTC patients with minimal extrathyroidal extension (mETE) and tumor diameter less than 4 cm were classified into T1-T2 stage, and there was also controversy about the treatment of central lymph nodes of these patients (7, 8). According to relevant studies, over 50% of patients with PTC have central lymph node metastases (CLNM) (9), and the majority of these patients are in the T1-T2 disease subgroup. Even in patients with clinically negative cervical lymph nodes (cN0), the rate of CLNM can range between 15.9% and 53% (10). Except for that, several studies still advocate CLNM is a risk factor for recurrence (11, 12). Considering the complications of CLND, such as laryngeal nerve injury and hypoparathyroidism, decision-making for these patients should better depend on individual patients rather than single approach recommended by guidelines. Based on preoperative clinical characteristics, several nomograms have been built to predict central or cervical LNM in PTC patients (13–16). However, there is no available nomogram based on the SEER database to predict CLNM in T1-T2 PTC patients without LLNM.

To that end, the present study used the preoperative clinical characteristics of T1-T2 PTC patients without lateral neck metastasis retrieved from the SEER database from 2010 to 2015 to construct a nomogram and assist clinicians in choosing the optimal treatment strategy for this particular patient group.

## Materials and methods

### Data source

Our data came from two databases. The training and internal verification data were extracted from the Surveillance, Epidemiology, and End Results (SEER) database, which is currently the largest publicly available cancer database in America, covering approximately 28% of the US population. Meanwhile, the external validation data were collected from the electronic medical record system of the Department of Thyroid Surgery in the First Hospital of China Medical University. All procedures performed in studies involving human participants were in accordance with the ethical standards of the Institutional Ethics Committee of China Medical University.

### Patients and variables selection

**Figure 1:**
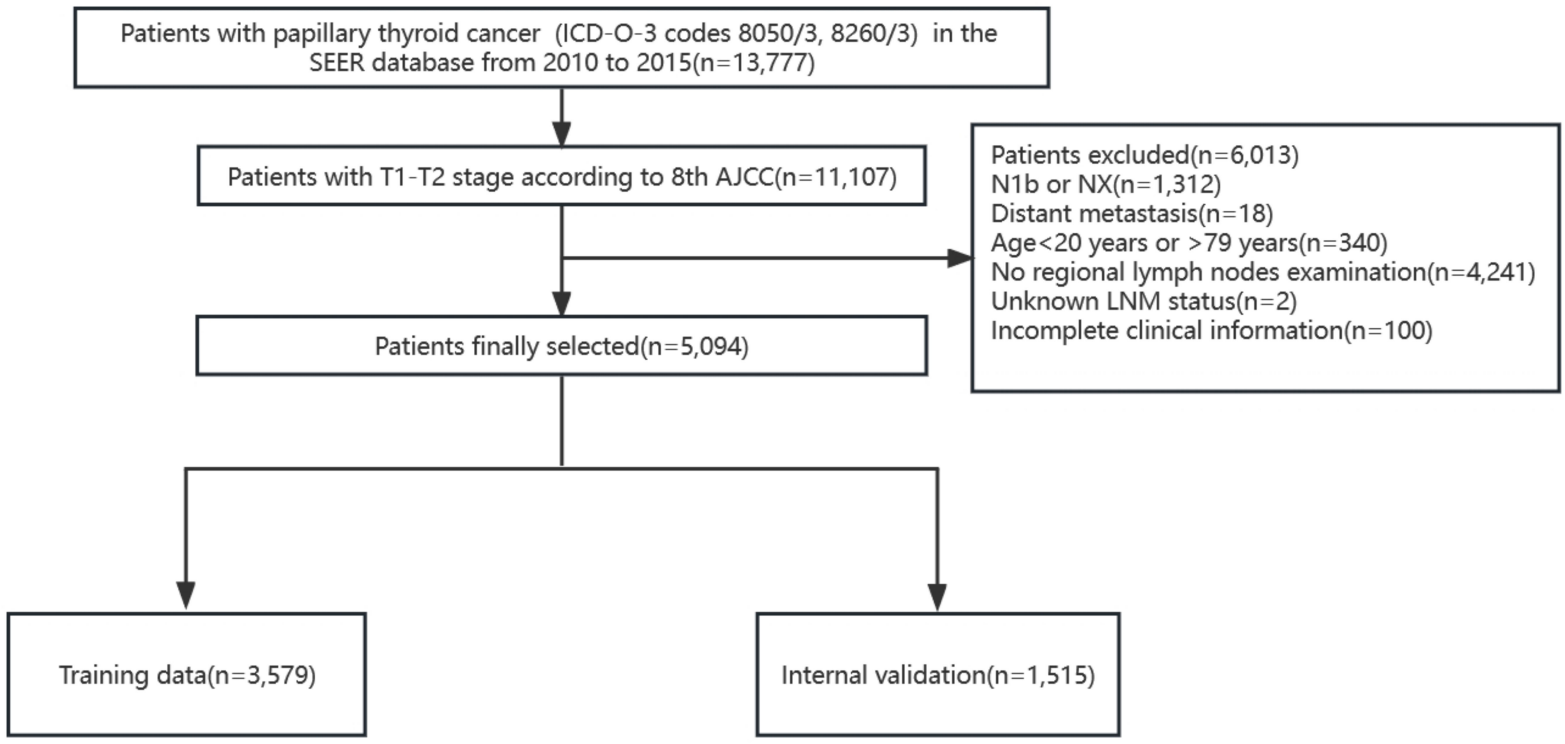
Flowchart of patient selection in SEER database. AJCC, American Joint Committee on Cancer.

According to the TNM staging of the 8th edition of AJCC, PTC patients (ICD-O-3 codes 8050/3, 8260/3) with T1 and T2 in the SEER database from 2010 to 2015 were firstly screened. The T stage in the 8th AJCC staging system was not available so we defined according to the tumor extension and tumor size. The exclusion criteria included patients with stages N1b or NX, with distant metastasis, age<20 years or >79 years, without regional lymph nodes examination record, unknown LNM status, and patients with missing clinicopathological characteristics of interest. A total of 5,094 patients were finally included and randomly divided into the training cohort (3,579 patients) and the internal validation cohort (1,515 patients) according to a 7:3 ratio (**Figure 1**). We extracted the following demographic information from the SEER database: age (years), gender (male or female), race (black, white or others), tumor size (≤1cm, >1cm and ≤2cm, >2cm and ≤4cm), minimal extrathyroidal extension (mETE) (code 450), central lymph node metastasis (N stage) and multifocality.

Simultaneously, we identified PTC patients undergoing thyroid surgery at the First Hospital of China Medical University from February to April 2021. The enrollment criteria were as follows: patients aged between 20 and 79 years, T1 and T2 patients with no lateral neck metastasis confirmed by preoperative evaluation (including ultrasonography, CT scan, and FNA) or postoperative pathology, and patients with unilateral lobectomy or total thyroidectomy with CLND. The exclusion criteria were as follows: patients with no lymph nodes examination, patients with distant metastases, patients with a history of thyroid surgery or concomitant head and neck cancer, and patients with incomplete or missing medical records. Finally, 300 patients were enrolled in the external validation cohort.

### Statistical analysis

The SEER*Stat software (version 8.4.0; https://www.seer.cancer.gov/seerstat) was used to screen patients from the SEER database. Initially, the receiver-operating characteristic (ROC) curve based on age-CLNM was plotted, and the maximum Youden index was determined using SPSS ver. 25.0 package (IBM Corp., Armonk, NY) as the optimal age cutoff. Next logistic univariate and multivariate regression analyses were used to screen risk factors that were associated with CLNM. Variables with a p-value < 0.05 from the univariate logistic regression were used for multivariate logistic regression to construct a risk prediction model nomogram. Harrell concordance indexes (C-index), equivalent to the area under the ROC curve, were calculated to evaluate the nomogram’s discrimination sensitivity. The calibration plot was used to visualize the comparison between the prognosis predicted by the nomogram and the actual outcome. Lastly, decision curve analyses (DCA) were performed to further assess the nomogram. All statistical analyses were performed using R software version 4.1.3.

## Result

### Clinicopathological characteristics

In this study, 5,094 patients (1,010 men and 4,084 women) were retrieved from the SEER database, and 300 patients (57 men and 243 women) were retrieved from the medical records of the First Hospital of China Medical University. Patients from the SEER database were randomly assigned to two groups in a 7:3 ratio: the training cohort (n=3,579) and the internal validation cohort (n=1,515). A third group (n=300) representing the external validation cohort was obtained from the First Hospital of China Medical University. Approximately 32.1% (1,149/3,579) of patients developed CLNM in the training group, while the internal validation group and external validation group reached 34.5% (522/1,515) and 34.3% (103/300), respectively. Next, continuous variables were processed, and the ROC curve of age against CLNM was plotted according to the training data. The maximum Youden index was calculated, and the best cutoff was 43.5 (Sensitivity 60.9%, Specificity 54.8%, Youden Index 0.158). The age variable was stratified into <44 and ≥44 (years) according to the results, and the tumor size was divided into ≤1, >1 and ≤2, >2 and ≤4 (cm) according to the T stage. The specific clinicopathological characteristics of the included patients are summarized in **Table 1**.

**Table 1:** Clinicopathological characteristics of T1-T2 PTC patients with no LLNM.

Variables	Subgroup	No. (%) of patients		
		Training data	Internal testing	External testing
		(n=3579)	(n=1515)	(n=300)
Age (years)	≥ 44	2011 (56.2)	846 (55.8)	154 (51.3)
	< 44	1568 (43.8)	669 (44.2)	146 (48.7)
Gender	Female	2875 (80.3)	1209 (79.8)	243 (81.0)
	Male	704 (19.7)	306 (20.2)	57 (19.0)
Race	Black	116 (3.2)	44 (2.9)	0
	Black	2935 (82.0)	1236 (81.6)	0
	Other*	528 (14.8)	235 (15.5)	300 (100)
Tumor size (cm)	≤ 1	1464 (40.9)	613 (40.5)	259 (86.3)
	> 1 and ≤ 2	1324 (37.0)	582 (38.4)	38 (12.7)
	> 2 and ≤ 4	791 (22.1)	320 (21.1)	3 (1.0)
Multifocality	Negative	2081 (58.1)	854 (56.4)	221 (73.7)
	Positive	1498 (41.9)	661 (43.6)	79 (26.3)
mETE	Negative	2998 (83.8)	1269 (83.8)	279 (93.0)
	Positive	581 (16.2)	246 (16.2)	21 (7.0)
CLNM	Negative	2430 (67.9)	993 (65.5)	197 (65.7)
	Positive	1149 (32.1)	522 (34.5)	103 (34.3)

Other* defined as the Asian/Pacific Islander and American Indian/Alaska Native.

PTC, papillary thyroid carcinoma; LLNM, lateral lymph node metastases; mETE, minimal extrathyroidal extension; CLNM, central lymph node metastasis.

### Univariate and multivariate analysis of CLNM variables

In the univariate analysis, age (p < 0.001), gender (p < 0.001), race (p < 0.001), tumor size (p<0.001), multifocality (p<0.001), and mETE (p<0.001), were found to be potential risk factors associated with CLNM (**Table 2**). Multivariate logistic regression modeling was further conducted to screen for significant variables associated with CLNM. The results were as follows: age < 44 years (OR= 1.825, 95% CI: 1.607-2.074, p<0.001), male gender (OR= 1.788, 95% CI: 1.534-2.084, p<0.001), white and other races (Asian/Pacific Islander and American Indian/Alaska Native) (white: OR= 2.635, 95% CI: 1.730-4.156, p< 0.001; other races: OR= 2.533, 95% CI: 1.625-4.074, p< 0.05), tumor size larger than 1 cm (1 cm<largest diameter ≤ 2 cm, OR= 2.458, 95% CI: 2.117-2.855, p<0.001; 2 cm<largest diameter ≤ 4 cm, OR = 3.119, 95% CI: 2.638-3.691, p<0.001), multifocality (OR= 1.328, 95% CI: 1.170-1.507, p < 0.001), and positive mETE (OR= 2.244, 95% CI: 1.911-2.637, p<0.001) (**Table 2**).

**Table 2:** Univariate and multivariate analysis of risk factors associated with CLNM.

Variables	Subgroup	Univariable		Multivariable	
		HR (95%CI)	P	HR (95%CI)	P
Age (years)	≥44	ref		ref	
	<44	1.766 (1.569, 1.990)	**<0.001**	1.825 (1.607, 2.074)	**<0.001**
Gender	Female	ref		ref	
	Male	1.733 (1.502, 2.000)	**<0.001**	1.788 (1.534, 2.084)	**<0.001**
Race	Black	ref		ref	
	White	2.436 (1.632, 3.775)	**<0.001**	2.635 (1.730, 4.156)	**<0.001**
	Other*	2.685 (1.757, 4.244)	**<0.001**	2.533 (1.625, 4.074)	**<0.001**
Tumor size (cm)	≤ 1	ref		ref	
	> 1 and ≤ 2	2.850 (2,470, 3.300)	**<0.001**	2.458 (2.117, 2.855)	**<0.001**
	> 2 and ≤ 4	3.824 (3.250, 4.500)	**<0.001**	3.119 (2.638, 3.691)	**<0.001**
Multifocality	Negative	ref		ref	
	Positive	1.450 (1.288, 1.634)	**<0.001**	1.328 (1.170, 1.507)	**<0.001**
mETE	Negative	ref		ref	
	Positive	2.568 (2.206, 2.989)	**<0.001**	2.244 (1.911, 2.637)	**<0.001**

Other* defined as the Asian/Pacific Islander and American Indian/Alaska Native. Bold values indicate statistical significance (p<0.05).

CLNM, central lymph node metastasis; HR, hazard ratio; mETE, minimal extrathyroidal extension.

### Nomogram for predicting CLNM in T1 and T2 PTC patients with no LLNM

**Figure 2:**
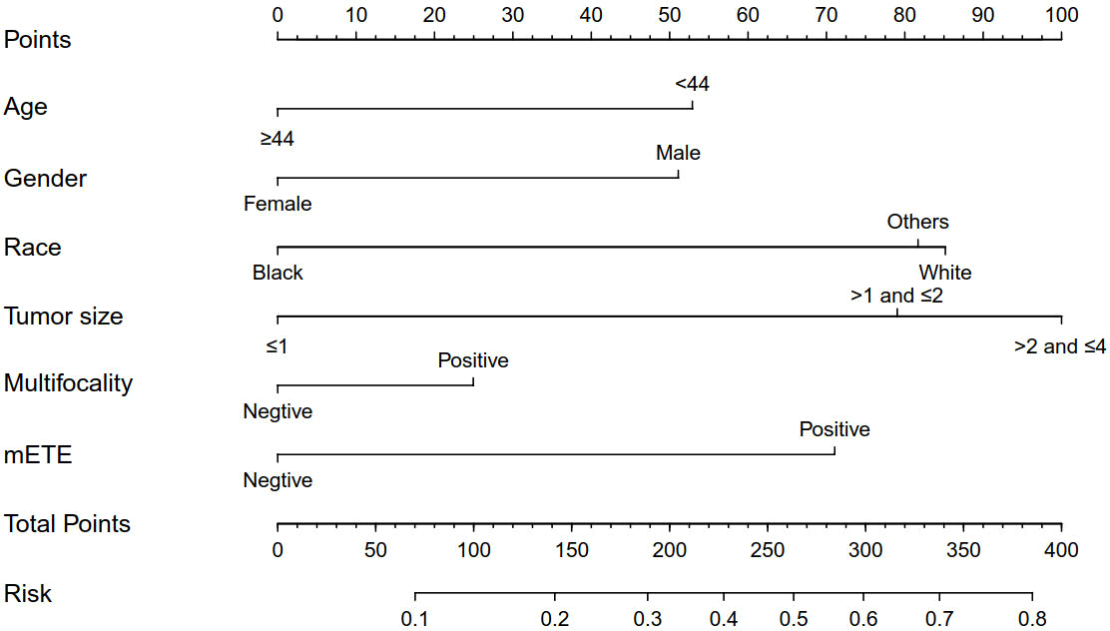
Clinicopathological characteristics-based nomogram used for prediction of CLNM in T1-T2 PTC patients with no LLNM. Other, Asian/Pacific Islander and American Indian/Alaska Native; mETE, minimal extrathyroidal extension; CLNM, central lymph node metastasis; LLNM, lateral lymph node metastases; Age (years); Tumor size (cm).

Based on the independent factors screened through multivariate analysis, a nomogram was established for predicting individual risk of CLNM. The risk of each factor, including age, gender, race, tumor size, thyroid capsule involved, and multifocality, was quantified in our predicting model (score of each factor was shown in **Figure 2**) to predict the presence of CLNM in T1-T2 PTC patients with no LLNM.

### Internal and external validation of the nomogram

**Figure 3:**
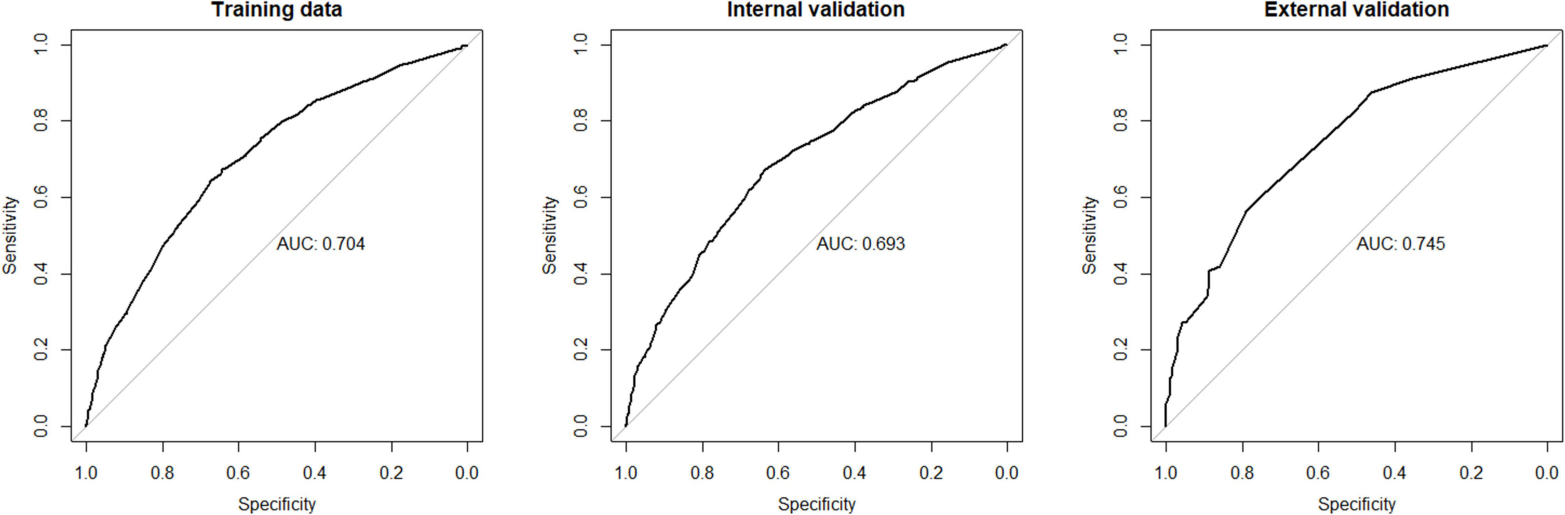
The receiver operating characteristics (ROC) curve and area under the ROC curve (AUC) in the training cohort, internal cohort, and external cohort. CLNM, central lymph node metastasis.

**Figure 4:**
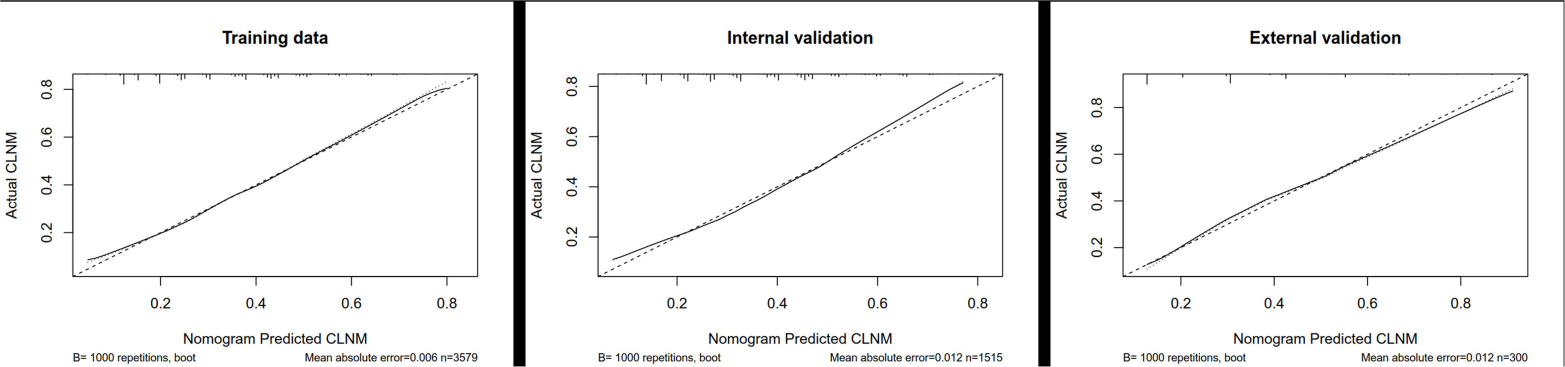
Calibration plot for our nomogram in the training cohort, internal cohort, and external cohort. CLNM, central lymph node metastasis.

Although we established a new nomogram for predicting CLNM with a robust C-index of 0.704, further validation was needed. Therefore, we developed an internal validation cohort using the SEER program and an external validation cohort using patients from our department. A C-index of 0.693 was achieved in the internal validation cohort, while a C-index of 0.745 was acquired in the external validation cohort, confirming our nomogram’s accuracy in predicting CLNM occurrence. The receiver operating characteristics (ROC) curve and area under the ROC curve (AUC) are shown in **Figure 3**. Furthermore, we also conducted a calibration plot for our nomogram, and a favorable agreement was found between the actual and estimated probability of CLNM (**Figure 4**).

**Figure 5:**
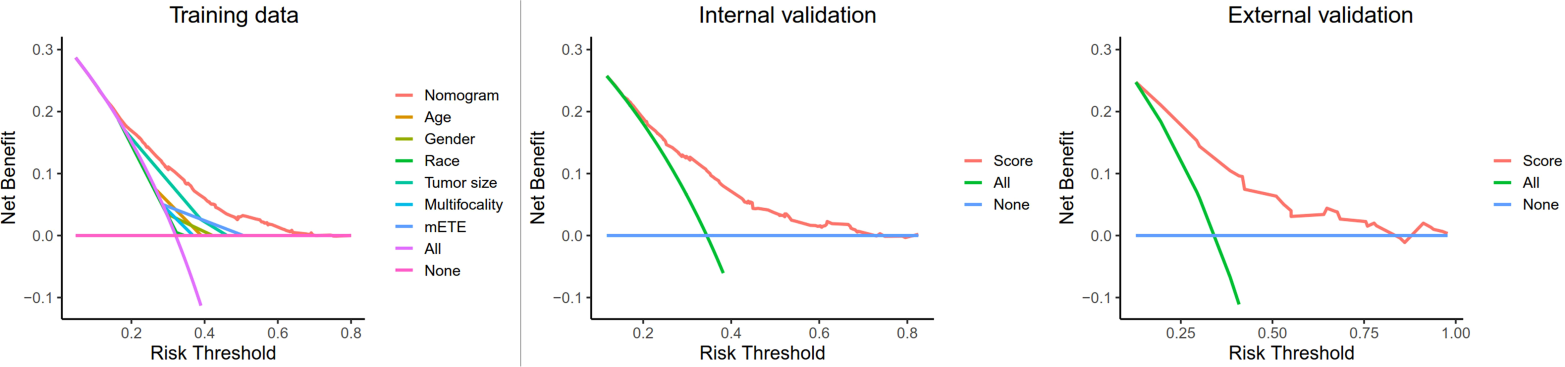
Decision curve analysis for CLNM in T1-T2 PTC patients with no LLNM in the training cohort, internal cohort, and external cohort. Net benefit = (true positives/N) − (false positives/N) * (weighting factor); Weighting factor = Threshold probability/(1−threshold probability); CLNM, central lymph node metastasis; LLNM, lateral lymph node metastases; mETE, minimal extrathyroidal extension.

Finally, a decision curve analysis (DCA) was designed to compare the predictive ability between the nomogram and the single-factor model. The standardized net benefits of the models were comparable, and there was a significant overlap between these models. Notably, the DCA revealed that the prediction ability of the nomogram was superior to that of the single-factor model in detecting CLNM in T1, T2 PTC patients with no LLNM. Furthermore, in the internal and external validation group, the nomogram was more effective than a treat-none or treat-all strategy when the threshold probability ranged from 0.2 to 0.7 (**Figure 5**).

## Discussion

The incidence of papillary thyroid cancer has increased yearly, mainly due to advances in diagnostic technology. Early-stage PTC tends to have a good prognosis, which dictates a conservative approach to diagnosis and treatment. According to the management guidelines of the ATA (2015), for adult patients with thyroid nodules and differentiated thyroid cancer, fine needle aspiration (FNA) was only recommended for patients whose maximum tumor diameter was larger than 10 mm combined with highly suspicious sonographic features (5). One retrospective multicenter study concluded that there was no significant difference in the risk of regional recurrence whether CLND was performed or not in cN0 PTC (17). In contrast, for some patients, incomplete lymph node dissection increases the rate of regional recurrence and repeated surgery (12). For T1-T2 PTC patients without LLNM in China, routine CLND is still recommended, which is not recommended for all such patients in guidelines of the ATA (2015) (5, 6). Additionally, for the change of the AJCC TNM classification, some T3 patients in the 7^th^ edition of AJCC TNM classification were reclassified into the T1-T2 stage, who were recommended a PCLND in the past (7, 8). In light of this, it is important to construct a visual predictive model to assist surgeons in deciding whether CLND is appropriate for these patients.

For low-grade, low-risk cN0 PTC patients, active surveillance (AS) and thermal ablation (AB) are also considered treatment modalities (18, 19). Considering the high incidence of CLNM in PTC, the long-term prognosis of these treatment options remains to be determined. According to Kuma Hospital, low-risk papillary thyroid microcarcinoma (PTMC) should be treated with AS instead of surgery, and patients with tumor diameters >1 cm do not always require immediate surgical intervention. Patients with older age, multifocality, and minimal invasion could also be targeted for AS (18). In a recent study by Sasaki et al., the need for conversion of AS to surgery for low-risk PTMC patients has decreased over time (20). However, consistent with previous studies and meta-analyses (21–23), our study found that age <44 years, multifocality, and mETE were risk factors for CLNM in small PTC (T1-T2) patients with no LLNM. Notably, there is a relatively high incidence of CLNM in cN0 patients (10), which led to some of these patients being treated with AS. To date, there are no studies on the long-term outcomes of patients treated with AS. Several studies (19, 24, 25) indicated that thermal ablation (TA) could be used as a treatment option for low-risk PTMC, but the longest follow-up time was 5 years. Considering that the incidence of CLNM is high, the impact on long-term prognosis is thus unclear. In our study, the CLNM rate of small PTC (T1-T2) with no LLNM was 32.1%-34.5%. Moreover, age, gender, race, tumor diameter, multifocality and mETE were considered risk factors for CLNM; therefore, AS or TA was probably unsuitable for high-risk patients in the prediction nomogram.

Previous studies have established several predictive models to predict CLNM in PTC patients. These had several limitations, such as a small sample size, the lack of external validation, or low racial heterogeneity. For instance, Gao et al. (13) collected information on clinicopathological characteristics and preoperative ultrasound images to predict CLNM in cN0 PTC patients. However, only 296 patients in a single center were included, and no external validation was performed. In a nomogram for predicting CLNM in PTC patients developed by Yang et al. (14), the internal and external validation C-indexes reached 0.857 and 0.825, and the model was significantly better compared to ultrasound; however, only 1,731 patients from China were included. One nomogram developed by Kim et al. (15) had a population size of 7,535 patients, but they were all of Korean descent. Furthermore, a recent analysis by Zhu et al. (26) in T1-T2, non-invasive, and cN0 PTC patients revealed that gender, tumor size and location, multifocality, age, and Delphi node status were independent predictors of CLNM. Moreover, by comparing the predictive ability of different types of models, a predictive model with an AUC of 0.70-0.75 was obtained. This was a single-center study that also lacked external validation. Feng et al. (16) previously used the SEER database to construct a nomogram for predicting cervical LNM in PTC patients and obtained excellent C-indexes of 0.716-0.733. There were also some studies, such as Min et al. (27) and Wei et al. (28), which have constructed excellent nomograms for predicting CLNM in different subgroup of PTC. And for T1-T2 with no LLNM, there were few studies. To our knowledge, we are the first to construct a nomogram based on the SEER program with internal and external validation cohorts for predicting CLNM. Moreover, we focused on T1-T2 patients with no LLNM, which was not studied in previous studies. To some extent, our nomogram may better predict CLNM in small PTC (T1-T2) patients, which is pretty common in clinical practice. As the restaging of T stage of the 8^th^ AJCC, some patients with mETE were identified as T1-T2. We also concluded that mETE was the risk factor of CLNM, and the HR was 2.568 (2.206, 2.989), which was not low. Additionally, we comprehensively validated our findings using ROC, calibration and DCA curves. Therefore, this nomogram could possess a higher predictive value in clinical practice. To make personalized decisions for patients, surgeons may use this nomogram independently of imaging examinations such as ultrasounds and CT scans.

Nevertheless, our study has some limitations. Firstly, due to the SEER database’s limitations, we could not include some variables, such as Hashimoto’s thyroiditis (HT), BRAFV600E mutation, several laboratory variables, and preoperative imaging features, which tend to associate with CLNM (13, 14, 29). Secondly, the external validation cohort is from a single center, and all patients were Chinese. Therefore, further external validation of this nomogram using patients from different countries and medical centers is warranted. Thirdly, this model was only designed for classic PTC patients, and was not applicable to other pathology types of PTC.

## Conclusion

Male gender, age <44 years, non- black population, tumor size larger than 1 cm, multifocality, and mETE were identified as risk factors for CLNM in T1-T2 PTC patients without LLNM. Although most T1-T2 PTC patients achieve long-term favorable prognoses, surgeons should reconsider whether neck lymph node dissection, TA and AS interventions are less suitable for patients at high risk of CLNM. Herein, using clinicopathological characteristics obtained from the SEER database, a nomogram was developed and validated for the prediction of CLNM in T1-T2 PTC patients without LLNM. Overall, this nomogram demonstrated robust effectiveness and could be used by clinicians to make personalized clinical decisions regarding T1-T2 patients.

## Data availability statement

The raw data supporting the conclusions of this article will be made available by the authors, without undue reservation.

## Author contributions

YS contributed to the conception of the study, performed the data analyses and wrote the manuscript. WS and JX helped perform the analysis with constructive discussions. HZ contributed to the conception and supervision of the manuscript. All authors contributed to the article and approved the submitted version.
