# Corrigendum: Nomogram for predicting central lymph node metastasis in T1-T2 papillary thyroid cancer with no lateral lymph node metastasis

**DOI:** 10.3389/fendo.2023.1148920

**Published:** 2023-02-01

**Authors:** Yubo Sun, Wei Sun, Jingzhe Xiang, Hao Zhang

**Affiliations:** Department of Thyroid Surgery, The First Hospital of China Medical University, Shenyang, China

**Keywords:** SEER (Surveillance Epidemiology and End Results) database, T1-T2, papillary thyroid carcinoma (PCT), central lymph mode metastasis, lateral lymph node metastasis, nomogram

In the published article, there was an error in **Figure 1** as published. Some data of this figure is not correct. The corrected **Figure 1** and its caption “Flowchart of patient selection in SEER database. AJCC, American Joint Committee on Cancer.” appear below.

**Figure 1:**
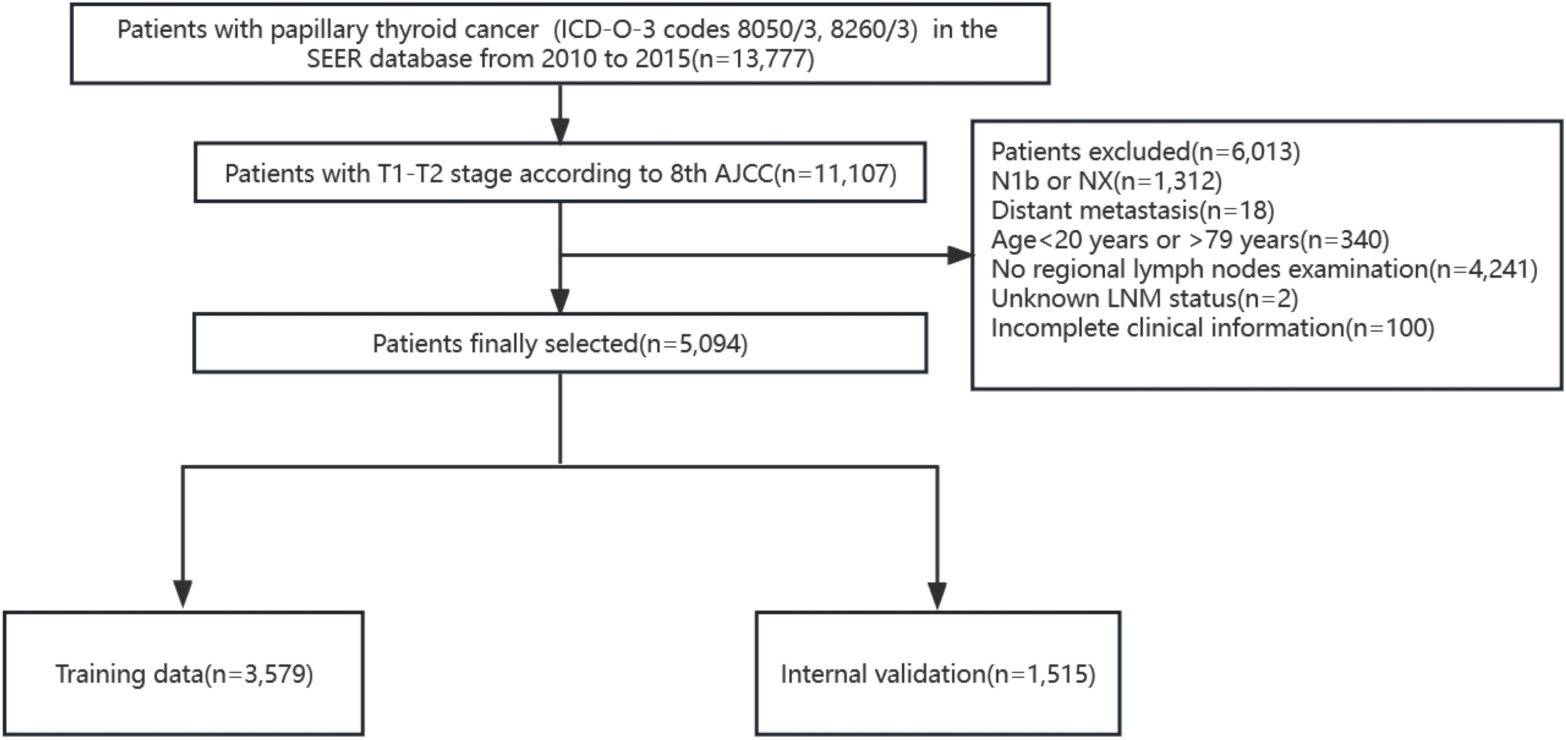
Flowchart of patient selection in SEER database. AJCC, American Joint Committee on Cancer.

The authors apologize for this error and state that this does not change the scientific conclusions of the article in any way. The original article has been updated.

In the published article, there was an error. Some data was not correct in the section of “Patients and variables selection”.

A correction has been made to Materials and methods, *Patients and variables selection*, Paragraph 1. This sentence previously stated:

“A total of 4,528 patients were finally included and randomly divided into the training cohort (3,173 patients) and the internal validation cohort (1,355 patients) according to a 7:3 ratio (**Figure 1**).”

The corrected sentence appears below:

“A total of 5,094 patients were finally included and randomly divided into the training cohort (3,579 patients) and the internal validation cohort (1,515 patients) according to a 7:3 ratio (**Figure 1**).”

The authors apologize for this error and state that this does not change the scientific conclusions of the article in any way. The original article has been updated.

